# Effects of a Hypocaloric Diet Plus Resistance Training with and Without Amino Acids in Older Participants with Dynapenic Obesity: A Randomized Clinical Trial

**DOI:** 10.3390/nu17030418

**Published:** 2025-01-23

**Authors:** Valentina Muollo, Andrea P. Rossi, Chiara Milanese, Valentina Cavedon, Federico Schena, Anna Giani, Silvia Urbani, Gloria Mazzali, Mauro Zamboni, Elena Zoico

**Affiliations:** 1 Department of Neuroscience, Biomedicine and Movement, University of Verona, 37131 Verona, Italy; valentina.muollo@univr.it (V.M.); chiara.milanese@univr.it (C.M.); valentina.cavedon@univr.it (V.C.); federico.schena@univr.it (F.S.); 2 Department of Medicine, Section of Geriatrics, Healthy Aging Center Treviso, 31100 Treviso, Italy; 3 Department of Medicine, Section of Geriatrics, University of Verona, 37100 Verona, Italy; annagiani92@gmail.com (A.G.); silvia.urbani@aovr.veneto.it (S.U.); gloria.mazzali@univr.it (G.M.); mauro.zamboni@univr.it (M.Z.); elena.zoico@univr.it (E.Z.)

**Keywords:** dynapenia, obesity, resistance training, supplementation, peak torque

## Abstract

Background/Objectives: Exercise and nutrition may be useful strategies in dynapenic and sarcopenic obesity management, but the identification of treatment modalities aimed at improving this condition is still lacking. We compared the effect of a five-month hypocaloric diet plus resistance training (RT) with and without essential amino acids (EAAs) on body composition, physical performance, and muscle strength among older adults with dynapenic obesity (DO). Methods: Older individuals (n = 48) with DO [(BMI ≥ 30 kg/m^2^ and/or high waist circumference and low handgrip strength (HGS)] were randomized into two double-blind groups (RT without EAAs vs. RT+EAAs). All participants followed a hypocaloric diet (1 g of proteins/kg spread over three meals) and RT for five months. Pre- and post-intervention assessments included the body composition (DXA), Short Physical Performance Battery (SPPB), HGS, one-repetition maximum (1-RM), and maximal isometric torque with an isokinetic dynamometer. Results: Both groups reduced body mass (RT: −4.66 kg; RT+EAAs: −4.02 kg), waist circumference (RT: −4.66 cm; RT+EAAs: −2.2 cm), total fat mass (RT: −3.81 kg; RT+EAAs: −3.72 kg), and compartmental fat mass with no between-group differences. Both groups improved 1-RM strength (33–47%), isometric torque for body mass (RT: 14.5%; RT+EAAs: 10.6%), and functional performance (chair stand (RT: −3.24 s; RT+EAAs: −1.5 s) and HGS (RT: −2.7 kg; RT+EAAs: 2.9 kg)) with no between-group differences. Conclusions: A moderate hypocaloric diet combined with RT improves body composition and physical function in DO participants, but EAA supplementation did not provide additional benefits.

## 1. Introduction

Recently, the concept of “dynapenic obesity” (DO), defined as the coexistence of low muscle strength and obesity, has been introduced [1,2].

The criteria for its definition, including low handgrip strength (HGS) and high body mass index (BMI) and/or high waist circumference, are mentioned in the review of the definitions and diagnostic criteria for sarcopenic and dynapenic obesity of the European Society for Clinical Nutrition and Metabolism (ESPEN) and the European Association for the Study of Obesity (EASO) [3,4]. However, a univocal and standardized definition for DO has not yet been achieved.

DO is associated with functional decline and is related to important unfavorable health outcomes such as the risk of worsening disability, hospitalization, and mortality in older adults [5,6,7], showing a greater risk for DO, in comparison with participants presenting dynapenia or obesity alone.

Only a few studies have focused on the effect of exercise and nutritional programs in participants with DO. They generally include relatively short interventions that typically last no longer than 12 to 16 weeks, mainly involving females, and show conflicting results [8,9,10,11]. To the best of our knowledge, previous studies in participants with DO have not examined how different interventions affect isometric strength, which is crucial for understanding the best strategies to counteract age-related muscle strength changes [12].

Moreover, there is a lack of studies testing the effect of amino acid supplementation in older adults with DO. Nutritional supplements may be important to avoid muscle loss associated with hypocaloric diets that reduce muscle protein synthesis and increase proteolysis. The optimal prescription of the amount of protein in the diet or the type of protein supplementation (essential amino acids (EAAs), branched chain amino acids (BCAs), or whey proteins) has not been characterized yet for DO [13]. In particular, EAA supplementation may be beneficial in older individuals with obesity, increasing amino acid availability for muscle protein synthesis and counteracting increased splanchnic extraction [14]. However, it is unknown if adding EAAs to a hypocaloric diet rich in protein may be beneficial in older adults with DO.

Therefore, this study aims to investigate the effects of a five-month hypocaloric diet that includes 1 g of proteins per kg spread in three main meals, combined with resistance training (RT) alone or associated with EAA supplementation, on anthropometric measurements, body composition, isometric maximal strength in both upper and lower limbs, and physical performance in older men and women with DO. Our hypothesis is that combining RT with EAAs could lead to further improvements in body composition, muscle function, and physical performance in older adults with DO. The primary outcome was the change from baseline in maximal isometric lower limb strength, evaluated using an isokinetic dynamometer over 5 months.

## 2. Materials and Methods

### 2.1. Study Design and Population

In this double-blind randomized controlled trial, a group of older men and women residing in Verona, Italy, was selected from patients of the Nutritional Clinic of Borgo Trento Hospital between March 2020 and February 2022. Inclusion criteria for the study required participants to meet the following conditions: (1) men and women aged between 60 and 80 years; (2) body mass index (BMI) of ≥30 kg/m^2^ and/or waist circumference ≥88 cm in women and ≥102 cm in males [15,16]; (3) the participants exhibited dynapenia based on HGS adjusted for the body weight ≤50th percentile from the NHANES population according to the sex- and age-specific cut-offs [4,17]; (4) participants resided in Verona (Italy) and (5) maintained stable weight in the previous 2 months; (6) they were previously sedentary (less than one hour of exercise per week in the last 6 months); and (7) they signed the informed consent for participation in the study.

Participants with the following specified conditions were excluded from the study: unstable angina or recent heart attacks, irregular or dangerous heart rhythms, heart failure beyond NYHA class II, severe respiratory failure, severe heart valve diseases like severe aortic stenosis, abdominal/thoracic aneurysms, recent intracerebral or subdural hemorrhages, poorly controlled high blood pressure, pacemakers or metal prostheses, severe chronic kidney failure, symptomatic musculoskeletal disorders, disk herniation, joint arthritis, acute joint injuries, recent hip/knee replacements (within the last six months), joint instability, large inguinal/abdominal hernias, acute retinal detachment/bleeding, recent eye surgery (laser, cataract, retinal, glaucoma), history of cancer (past 5 years), dementia diagnoses, and eating disorders. The Ethical Committee of the University of Verona approved the study with Protocol Record Number 1956CESC (Approval date: 28 November 2018) and it was registered at ClinicalTrials.gov with Identifier NCT05938205, submitted on 21 February 2020. Written informed consent was obtained from the patients to publish this paper.

**Figure 1:**
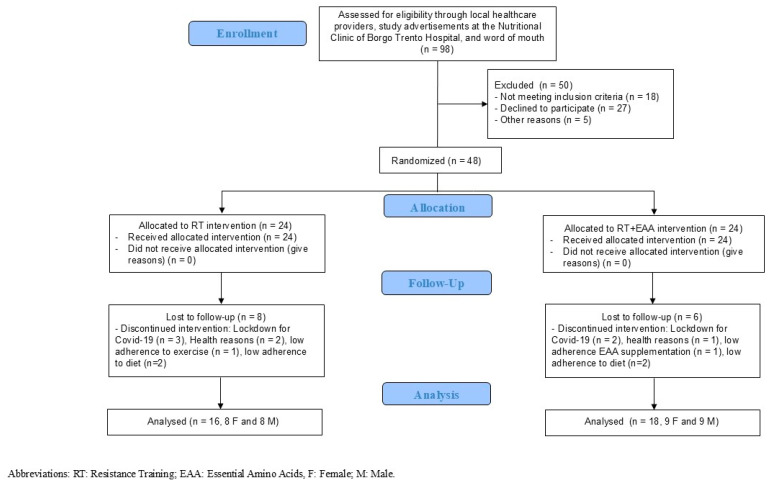
Flow chart of intervention.

Forty-eight participants were randomly allocated by the supervisors (APR and EZ) to one of the two groups and stratified according to sex in order to ensure equal gender representation across study groups and followed for 5 months. The five-month length of the nutritional and physical exercise intervention was established based on previous published data of clinical trials conducted in sarcopenic obese patients [18]. Fourteen participants were lost at follow-up. Therefore, the final analysis was performed on 34 participants with DO (17 women and 17 men). The complete flow chart of our study is presented in Figure 1.

### 2.2. Anthropometric and Body Composition Assessment

With the participants wearing light indoor clothes and no shoes, body weight was measured to the nearest 0.1 kg (Salus scale, Milan, Italy) and height to the nearest 0.5 cm using a stadiometer (Salus stadiometer, Milan, Italy) as described elsewhere [19]. The BMI was calculated as weight (kg) divided by height (m) squared [19]. Each measurement was taken once. Waist circumference was obtained with a measuring tape as the minimum circumference between the xyphoid process and the umbilicus [19]. To ensure accuracy, waist circumference was measured three times for each participant, and the average of these measurements was used for the analysis.

For a comprehensive evaluation of total and regional body composition, including lean mass and body fat, we utilized dual-energy X-ray absorptiometry (DXA) and a full-body scan (QDR Horizon W, Hologic, MA, USA; Fan-Bean Technology, ver. 12.4.2) following the manufacturer’s recommended procedure. To further refine our analysis, we computed appendicular lean mass (ALM), which is the sum of lean mass in the arms and legs [20], and ALM normalized to BMI (ALM/BMI).

### 2.3. 1-RM Assessment

Before the commencement of the training intervention, participants underwent three familiarization sessions aimed at acquainting them with resistance isotonic machines. During this period, correct lifting techniques and appropriate breathing methods were provided and practiced using both submaximal and near-maximal loads. After this familiarization phase, one-repetition maximum (1-RM) was assessed in each participant and the evaluations were conducted at baseline, before each month’s initial training session, and after a 5-month intervention period.

The 1-RM evaluation protocol involved a ten-minute full-body warm-up phase, followed by eight repetitions with a light load, which was intended to serve as a specific warm-up. Subsequently, the load was progressively increased until the participant reached failure after completing 3–6 repetitions. This range of repetitions was appropriate for individuals lacking prior strength training experience [21,22]. Emphasis was placed on adhering to a proper lifting technique, devoid of compensatory movements or assistance. The Brzycki formula [23] was employed to estimate 1-RM based on the outcomes of the strength testing. For each participant, the heaviest successful lift was determined following a maximum of five attempts, with 2 min rest intervals implemented to facilitate recovery.

The initial estimated 1-RM was determined for six distinct exercises: a chest press, leg press, lat machine, triceps pushdown, leg extension, and vertical traction machine. Estimated 1-RM values were tracked monthly for chest press, leg press, and lat machine exercises. Importantly, the updated 1-RM values obtained at the beginning of each month were used to reprogram the training loads. This approach ensured that the weight increments (from 75% to 80%) were adjusted based on participants’ progress rather than the baseline 1-RM values. However, for the triceps pushdown, leg extension, and vertical traction machine exercises, the 1-RM values were adjusted based on the baseline 1-RM value.

### 2.4. Handgrip Strength and Physical Performance Assessments

Upper body strength was evaluated by using a handgrip dynamometer (CAMRY, Digital Hand Dynamometer model: EH101, South El Monte, CA, USA). Participants underwent a five-minute familiarization period before the actual assessment. The HGS was recorded while the individual stood upright with their arm, forearm, and wrist maintained in a neutral alignment [24]. Participants were instructed to exert maximal handgrip pressure for 5 s. Three attempts were made using the dominant arm, each separated by a 1 min rest interval. The best trial regarding absolute HGS (measured in kilograms) and adjusted by body weight was selected for the subsequent statistical analysis [17].

Physical performance of the lower extremities was assessed using the Short Physical Performance Battery (SPPB) test, including gait speed, a chair stand repeated 5 times, and balance tests [25]. For each administered test, an experimenter employed a stopwatch to measure the time taken, who then assigned a score ranging from 0 to 4 points based on recommended cut-off values [26]. This cumulative process resulted in a potential total of 12 points as the maximum attainable score.

### 2.5. Knee Extensor Muscle Torque Measurements and Analysis

The knee extensors’ maximal isometric torque was assessed at baseline and at the 5-month follow-up using the CMSi Cybex Humac Norm Dynamometer (Lumex, Ronkonkoma, NY, USA). After a familiarization period, three maximal isometric trials were conducted at four different knee angles, 30°, 60°, 75°, and 90°, with a 60 s recovery between each trial. The highest isometric peak recorded at each tested angle was used for the subsequent analysis. Additionally, the maximal torque (Tmax) and optimal angle were calculated as previously reported [27].

### 2.6. Nutritional Intervention

Participants adhered to a moderately low-energy diet with the goal of a 5% reduction in initial body weight at the end of the study. A 24 h dietary recall interview to assess the dietary habits of each subject was performed by a trained dietician and analyzed with specific software, at the beginning of the study. Nutritional intervention was tailored for each participant, providing a diet of 500 kcal below their resting energy expenditure, as determined by the Mifflin–St. Jeor formula and adjusted by a physical activity level of 1.4 [28]. Nutritional recommendations were provided to each participant to ensure 1 g of proteins per kilogram of body weight with a diet composed of 60–50% carbohydrates, 25% fat, 15–25% of protein, and 20 g of dietary fiber. These provisions were spread across three main meals and two snacks. As this was not a controlled feeding study, participants did maintain some autonomy over their diet but were monitored and counseled by the dietician that every month checked for dietary compliance.

During the treatments, 2 patients for each arm of the study (8%) dropped out for low compliance to nutritional recommendations, with an overall high adherence to the nutritional program in the entire study (92%). Moreover, patients with weight loss at the end of the study lower than 3% were excluded from the final analysis. We decided to exclude them from the final analysis because those subjects were not adherent to the prescribed hypocaloric diet. Low levels of weight loss, such as a 0–2% reduction in body weight, can lead to small improvements in health outcomes (particularly physical performance and muscle strength) that may not reach statistical significance, particularly in small studies [29].

### 2.7. Supplements (Amino Acids or Placebo)

Participants were randomly assigned to one of two double-blind groups: one group received an isocaloric product containing maltodextrins (placebo), while the other group received 8 g/day of EAAs (as suggested in the instructions of the commercially available product) (2 packages of 4 g of EAAs distributed as follows: L-Leucine, 1.25 g; L-Lysine, 0.650 g; L-Isoleucine, 0.625 g; L-Valine, 0.625 g; L-Threonine, 0.350 g; L-Cysteine, 0.150 g; L-Histidine, 0.150 g; L-Phenylalanine, 0.100 g; L-Methionine, 0.050 g; L-Tyrosine, 0.030 g; and L-Tryptophan, 0.020 g; Amino-Ther, Professional Dietetics, Milan, Italy). We used the dosage of the commercially available supplement, based on positive results obtained in previous studies even if conducted in different populations (i.e., patients with malnutrition, sarcopenia, etc.). Participants were instructed to consume one packet of amino acids (or placebo) in the morning and another in the afternoon, dissolved in half a glass of water, away from meals. On days of exercise, participants ingested the supplement (amino acids or placebo) an hour before the commencement of the training session [30]. Adherence to the supplementation (either to placebo or amino acids) was checked weekly before the exercise session by an exercise physiologist blinded to the randomization, who received the empty boxes of the supplements, checking for the patient’s compliance with the supplementation and eventual side effects. Side effects rarely consisted in gastrointestinal discomfort that passed over time, ingesting the supplementation after a light snack, and being already present in the nutritional program of the obese patient, and only 1 patient exited the study for low adherence to EAA supplementation (Figure 1). In the placebo + RT group, only 1 patient dropped out of the study because of poor glycemic control (Figure 1, health reason for 1 out of 2 patients).

### 2.8. Progressive Resistance Training Protocol

All participants were engaged in a progressive RT regimen lasting five months, with sessions conducted three times a week, each lasting for an hour under the supervision of a certified exercise physiologist. All participants followed a uniform RT protocol performed at the Sport and Exercise Sciences facility at the University of Verona, specifically within the Department of Neurosciences, Biomedicine, and Movement Sciences. The training program began with three sets of 8 repetitions during the first two weeks of each month, progressing to 10 repetitions during the third and fourth weeks, performed at 70% of their one-repetition maximum (1-RM) in the first month. Subsequently, the intensity progressively increased to 75% of 1-RM during the second and third months, followed by a sustained intensity of 80% 1-RM for the remainder of the intervention period.

Each training session comprised a 10 min warm-up phase encompassing aerobic activities, mobility exercises, and balance drills. The central phase, lasting 40 min, included six isotonic machine-based exercises targeting both upper and lower body muscle groups. These exercises included the leg press, leg extension, lat machine, chest press, triceps pushdown, and vertical traction, with approximately 6–7 min allocated to each, including both execution time and rest intervals. In addition, body weight exercises and elastic band exercises were incorporated into some sessions to complement the isotonic machine exercises, offering variety and targeting stabilizing muscle groups. Participants were instructed to execute both concentric and eccentric phases of each exercise within 2–3 s, with rest intervals of 1–2 min between sets. No participants experienced adverse events associated with the resistance training intervention.

Adherence to the regimen, quantified by the number of completed exercise sessions, was closely monitored individually. To be considered for the analysis, participants were required to attain an attendance rate exceeding 70% by the end of the intervention.

### 2.9. Statistical Analysis

All statistical analyses were conducted using IBM SPSS software version 28 (Chicago, IL, USA). For a randomized trial with a superiority analysis, we estimated that a sample size of 23 participants per group, which allowed for 20% dropout, would provide >80% power to detect a difference between groups in the chair-rising test, assuming a mean between-group difference of 1.78, with a pooled SD of 1 at an α level of 5%. We considered 2.6 s as the margin of clinically relevant difference in the chair-rising test, as demonstrated in a population-based study of community-dwelling elderly individuals over a 3-year follow-up period [31].

Data are reported as the mean ± 95% confidence interval unless otherwise indicated. The sample size, while sufficient to assess the primary outcomes, limited our ability to conduct robust subgroup analyses without risking statistical power loss and interpretability of results and for this reason, we chose not to stratify the results by age and sex. Baseline characteristics among the two groups were compared using one-way ANOVA and chi-square tests. Normal distribution (assessed via Shapiro–Wilk’s test), homogeneity of variances (assessed by Levene’s test), and sphericity (checked using Mauchly’s test) were verified for all variables (*p* > 0.05). Paired *t*-tests were utilized for intragroup change examination. To assess differences between groups in anthropometric measurements, body composition, muscle strength, and overall physical performance outcomes, an analysis of covariance (ANCOVA) was employed, with baseline values as covariates. Post hoc pairwise comparisons were conducted using the Bonferroni method.

A two-way ANOVA for repeated measures was applied to identify interactions between time and groups in order to evaluate 1-RM. In cases of significant main effects or interactions, the Bonferroni post hoc test was employed. The significance threshold was set at *p* ≤ 0.05.

## 3. Results

### 3.1. Characteristics of Participants and Training Compliance

The anthropometric and clinical characteristics of participants in the two intervention groups, RT and RT+EAAs, are presented in Table 1. No significant differences were observed in the two groups at baseline (*p* > 0.05). In particular, sex and the prevalence of sarcopenia were similarly distributed between the two groups. No difference in the prevalence of diabetes and hypertension was also observed.

Participants who completed the intervention showed a high adherence with an average attendance rate of 86% (RT: 85.9 ± 8.9%, 95%CI [81.6–90.3]; RT+EAAs: 85.5 ± 9.1%, 95%CI [81.3–89.7]), with no differences between the two groups (*p* = 0.879, in tables).

### 3.2. Changes in Anthropometric, Body Composition, and Functional Tests

No differences were found in anthropometric, body composition, and functional tests between the two groups at baseline (Table 1).

After the 5-month intervention, the ANCOVA showed no significant differences between interventions for the examined variables (Table 2). In particular, compared to baseline, both groups reduced body weight, BMI, waist circumference, and arm and leg fat mass (*p* < 0.05). Conversely, lean mass showed a slight non-significant decrease in the RT group and was preserved in the RT+EAA group; no changes were observed in ALM, whilst ALM/BMI improved in both groups. Only two participants transitioned from dynapenic to non-dynapenic obesity (one in each group). Sarcopenic obesity decreased from 31.3% to 25% in the RT group and from 55.6% to 50% in the RT+EAA group, resulting in an overall reduction of 5.5 percentage points.

Regarding the functional tests, both groups improved in the chair stand, the SPPB total score, and the HGS, even after adjustment for body weight (*p* < 0.05). Specifically, 13 participants (38%) transitioned from being dynapenic at baseline to non-dynapenic post-intervention, with a higher prevalence observed in the RT+EAA group (44%) compared to the RT group (31%). No between-group effect was observed in the changes in considered variables.

### 3.3. Changes in 1-RM in the Isotonic Machines

**Figure 2:**
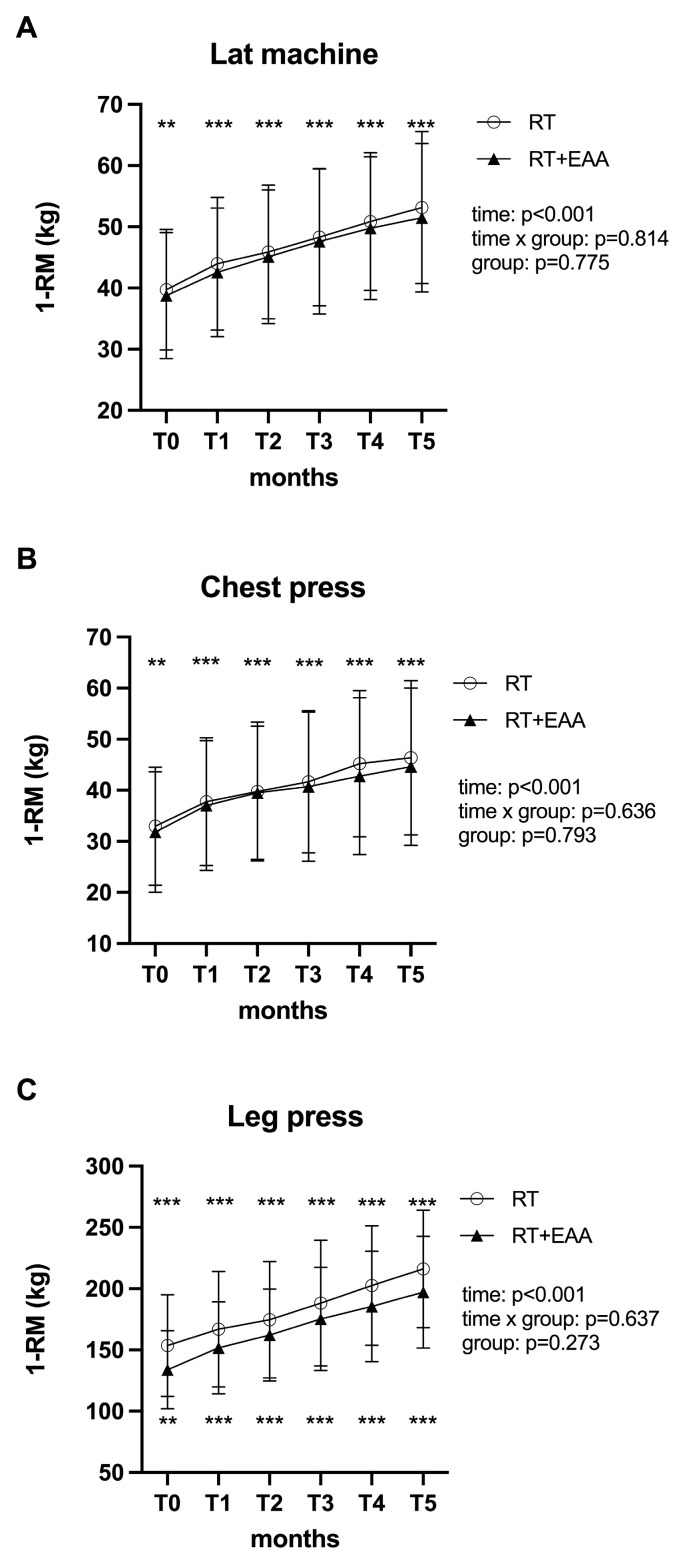
Changes over time in the estimated one-repetition maximum test. Figure Legend: The lat machine (panel **A**), chest press (panel **B**), and leg press (panel **C**) are presented in the RT (○) and RT+EAA (▲) groups. *** *p* < 0.001 indicates a significant difference compared with baseline. ** *p* < 0.01 Abbreviations—RT: Resistance Training; EAA: Essential Amino Acid; 1-RM: One-Repetition Maximum.

Figure 2 illustrates the monthly improvements on isotonic machines tested before each training session. There was no significant interaction between the two intervention groups for the lat machine (panel A), chest press (panel B), and leg press (panel C). However, in both groups, the main effect of time showed a statistically significant improvement in the performance on all three tested isotonic machines (all *p* < 0.001).

In comparison to baseline, similar improvements were observed in both groups on the lat machine (RT: 34% and RT+EAAs: 33%, all *p* < 0.001), chest press (RT: 41% and RT+EAAs: 40%, all *p* < 0.001), and leg press (RT: 47%, *p* < 0.001 and RT+ EAAs: 47%, all *p* < 0.001).

### 3.4. Changes in Knee Extensor Isometric Torque at Different Angles

**Figure 3:**
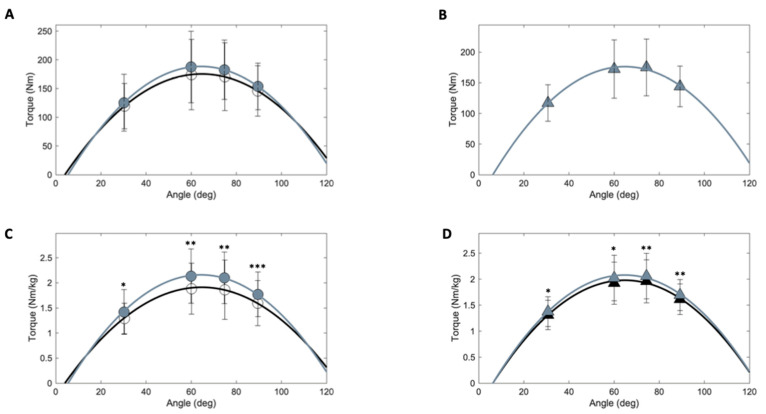
Changes in the knee extensor torque–angle relationship. Figure Legend: Pre- (black line) and post- (gray line) isometric knee extensor torque at different knee angles for the RT group (panels **A**,**C**, circles) and the RT+EAA group (panels **B**,**D**, triangles). The values are expressed in absolute units (panels **A**,**B**) and normalized for body mass (panels **C**,**D**). * *p* < 0.05, ** *p* < 0.01, and *** *p* < 0.001 indicate a significant difference compared to baseline.

The torque–angle relationship of the knee extensors after the intervention is shown in Figure 3. After the 5-month intervention, the ANCOVA showed no differences between groups (Table 3). Compared to baseline, Tmax expressed in absolute units increased only in the RT group (8.5%), while Tmax normalized for body mass increased in RT and RT+EAA groups (14.5% and 10.6%, respectively), whereas at the different knee angles, both groups similarly increased the torque normalized for body weight (*p* < 0.05).

## 4. Discussion

Our study shows that a moderate hypocaloric diet, providing 1 g of proteins per kg spread across three main meals, combined with RT, determines body weight and fat mass loss with fat-free mass preservation as well as significant functional improvement in older adults with DO. At this dose, and in this population, amino acid supplementation, along with a balanced hypocaloric diet providing 1 g of proteins per kg and RT, did not show any additional effect on body composition, physical performance, and muscle strength in participants with DO. These results may indicate that the amount of EAAs prescribed might not induce further response in muscle protein synthesis, as compared with a balanced hypocaloric diet, with 1 g of proteins per kg of body weight, and RT alone in DO older adults.

Unfortunately, there is a lack of studies testing the effect of amino acid supplementation with or without physical exercise, in particular in older adults with DO. Only a few studies were previously conducted in sarcopenic adults with obesity, with shorter lengths of intervention and similar-to-lower content of EAAs associated or not with other supplements and physical exercise compared to our study [32,33,34]. These studies did not show greater effects of nutritional supplementation on body composition than exercise plus a hypocaloric diet rich in protein alone, at least in older adults with sarcopenic obesity [32,33,34]. Moreover, the lack of differences observed in our study in the two weight loss groups even with a relatively larger amount of EAA supplementation (i.e., eight grams of amino acids) supports the futility of any EAA supplementation when patients have correct amounts of proteins in their hypocaloric diet combined with RT, which is in line with the Statement of the American Society for Nutrition and Obesity [35].

In our study, an improvement in the chair stand test, SPPB, and peak HGS in the two weight loss groups was also observed. These results are in line with previous studies and meta-analyses [8,32,36,37] but in contrast with Vasconcellos et al., who evaluated the impact of 10 weeks of RT on muscle strength and physical performance in women with handgrip ≤ 21 kg, finding no changes in SPPB or muscle strength [9]. The discrepancies in results can likely be attributed to differences in the type of intervention, duration, and intensity of the resistance exercise program, and the characteristics of the study populations. For instance, Vasconcellos et al. [9] investigated a group of 28 women with obesity aged 65 to 80 years old, using dynapenia cut-offs that differed from those in our study, as theirs were not normalized for BMI. Moreover, their intervention consisted solely of a 10-week resistance exercise program [9], which was shorter than ours (10 weeks with twice-weekly sessions) and conducted at a lower intensity (40–60% vs. 70–80% of 1-RM). Unlike our study, their intervention did not include a nutritional program, which may further explain the observed differences.

In parallel with the pattern observed for physical performance, HGS exhibited improvement post-intervention in both groups, with no significant distinctions between treatments. HGS is the most commonly employed metric for assessing muscle strength [38]. In parallel with the pattern observed for physical performance, HGS exhibited significant improvement post-intervention in both groups, with no discernible distinctions between the treatment groups. This suggests that RT, with or without EAA supplementation, is effective in improving muscle strength in older adults with dynapenic obesity. Interestingly, approximately 38% of participants transitioned from being dynapenic to non-dynapenic following the intervention. This aligns with previous findings that RT improves HGS in older adults, as also reported by Hsu et al. in their meta-analysis [39]. Importantly, the gradual progression in training intensity, starting at 70% 1-RM and advancing to 80%, may have facilitated these improvements without adverse events.

Interestingly, upper and lower body strength, as measured by monthly 1-RM assessments, exhibited similar gains in both study groups. In a meta-analysis involving healthy older adults [40], there was a collective improvement in 1-RM for lower limbs, favoring individuals who received both protein supplementation and exercise compared to those who underwent exercise alone. However, only limited studies with conflicting results in populations with DO have employed 1-RM as an outcome for assessing maximal strength gains [10,11]. Our results are in line with those of Carvalho et al., who, in a population of men with DO, found an improvement in lower limb muscle strength, after 12 weeks of mixed power training without a weight loss program [11]. Their 1-RM protocol used incremental loads to determine the maximum weight that could be lifted once, differing slightly from our Brzycki-based estimation method. Similarly, the MONET study, involving 31 postmenopausal women with DO with a 6-month follow-up, showed an additive beneficial effect of RT combined with caloric restriction on muscle strength, as evaluated with the leg press [10].

Nabuco et al., in a population of patients with DO, found no differences in chest press and leg extension 1-RM between groups receiving exercise plus whey protein supplements and those receiving only exercise, finding lower improvements in 1-RM as compared to our study [34]. Their method directly measured the 1-RM as the heaviest successful lift, contrasting with our submaximal effort estimation approach, which may partly explain the differences. Furthermore, neither group in Nabuco’s study underwent a weight loss program, which may have influenced the observed results.

To the best of our knowledge, this is the first study to examine the torque–angle profile in participants with DO. Both groups showed significant improvements in maximal knee extensor torque–angle relationships after adjusting for body weight, though not in absolute terms. These findings align with Sénécal et al., who reported no gains in leg maximum isometric strength of knee extensors after a 12-week RT intervention in women with DO [8]. The lack of strength improvements may be due to differences between the training modality and the isokinetic strength test. However, adjusting for body mass, a key mobility indicator [27], revealed a 6–14.5% improvement, highlighting the relevance of weight-adjusted strength in interventions involving weight loss.

Overall, the results of this study suggest that individuals with DO have a remarkable capacity to adapt to prescribed exercises, and that amino acid supplementation may not provide additional benefits compared to RT alone. Our study did not include a group that performed only RT, and the existing literature demonstrates that RT alone can lead to substantial benefits on muscle strength and physical performance [41,42]. Therefore, we can only hypothesize that an intense RT regimen may be sufficient on its own to drive significant improvements. Moreover, considering the three variables (i.e., weight loss, protein intake, and RT), we cannot exclude the possibility that the quantity of protein provided might also be important when combined with such intensive RT.

A recent review on the effects of nutritional supplementations in sarcopenic adults found that administering leucine in combination with a suboptimal protein or essential amino acids before RT is an effective strategy to counteract anabolic resistance in aged muscle, thereby preserving or increasing muscle mass and strength [43]. Additionally, we cannot rule out the potential benefits of a high-protein hypocaloric diet combined with RT for older dynapenic individuals with obesity. However, to our knowledge, there are no published data specifically addressing the effects of a high-protein diet in sarcopenic or dynapenic obese populations. Other dietary approaches such as intermittent calorie restriction, the Mediterranean diet, and high-protein diets are currently under investigation [44]. A recent literature review recommends that older adults consume higher quantities of high-quality protein per meal (1.0–1.2 g/kg/d) or low-dose leucine-enriched proteins (at least 3 g/day), ideally paired with lower protein doses (10–20 g protein/day) and combined with RT shortly after the meal for optimal benefits [45].

Finally, given the novelty of these findings (i.e., the type of exercise proposed, the rigorously increased progression and monitoring of intensity, and the functional evaluation employed, and the general lack of existing literature on this population), further research to evaluate the separate effects of a hypocaloric diet, protein intake, and RT alone is necessary to confirm and expand upon these results.

Several limitations warrant a mention. Primarily, the relatively small sample size could determine a lower statistical power. The slow recruitment process, spanning two years and occurring amidst COVID-19 lockdowns, posed a significant challenge. Moreover, the high dropout rate, influenced by the pandemic and consequent low intervention compliance, further compounded this limitation. Secondly, we were unable to evaluate the isolated effects of RT or EAA, as the study design did not include groups receiving only RT or only EAA. Despite these limitations, our study is strengthened by the use of a randomized controlled design to examine the effects of RT and amino acid supplementation in a population of older men and women with DO. Moreover, in contrast with previous intervention studies in DO, both sexes were well represented, and the study duration was much longer, considering that most of the similar intervention studies were limited to 10 to 12 weeks and generally included postmenopausal women only. Lastly, advanced and validated techniques were used to measure isotonic and isometric strength, and the adherence to a progressive supervised RT regimen lasting five months (three times a week) was high.

DO is easier to diagnose in clinical practice, compared with sarcopenic obesity, but similar studies involving participants with sarcopenic obesity should be replicated in order to test the additional effect of amino acid supplements in weight loss programs combined with RT in this population.

## 5. Conclusions

A moderate hypocaloric diet, including 1 g of proteins per kg spread across three main meals, combined with RT exercise determines body weight and fat mass decreases as well as significant functional improvement, with fat-free mass preservation in DO participants. Amino acid supplementation does not seem to add any additional benefit for this population.

## Figures and Tables

**Table 1:** Baseline characteristics of participants.

Outcomes	RT (n = 16)	RT+EAAs (n = 18)	*p* Value
Men (n, %)	8 (50.0%)	9 (50.0%)	1.000
Overweight (n, %)	4 (25.0%)	5 (27.7%)	1.000
Age (years)	65.75 ± 3.94	66.67 ± 3.85	0.932
Height (m)	1.67 ± 0.08	1.66 ± 0.07	0.983
BMI (kg/m^2^)	33.09 ± 4.07	32.37 ± 3.72	0.441
Waist circumference (cm)	109.5 ± 11.04	105.83 ± 9.45	0.305
Comorbidities			
Diabetes mellitus (n, %)	2 (12.5%)	2 (11.1%)	1.000
Hypertension (n, %)	11 (68.8%)	9 (50.0%)	0.315
Myocardial infarction (n, %)	1 (6.3%)	1 (5.6%)	1.000
Hypercholesterolemia (n, %)	9 (56.3%)	6 (33.3%)	0.300
Sarcopenia			
Prevalence (n, %)	5 (31.3%)	10 (55.6%)	0.185

**Table 2:** Anthropometric, body composition, and clinical tests and functional tests after 5 months of intervention.

All Groups	RT (n = 16)		Within-Group PRE vs. POST	RT+EAAs (n = 18)		Within-Group PRE vs. POST	Between-Group PRE	Between-Group PRE vs. POST
Outcome	Baseline	Mean Change (95% CI)	*p* Value	Baseline	Mean Change (95% CI)	*p* Value	*p* Value	*p* Value
BW (kg)	91.98 ± 13.6	−4.66 (−6.06 to −3.25)	<0.001	88.89 ± 12.57	−4.01 (−5.69 to −2.43)	<0.001	0.824	0.748
BMI (kg/m^2^)	33.09 ± 4.07	−1.71 (−2.25 to −1.16)	<0.001	32.37 ± 3.72	−1.48 (−2–04 to −0.92)	<0.001	0.441	0.699
WC (cm)	109.5 ± 11.04	−4.62 (−6.93 to −2.31)	0.001	105.83 ± 9.45	−2.25 (−4.36 to −0.13)	0.039	0.305	0.117
Arm fat (kg)	4.63 ± 1.78	−0.30 (−0.53 to 0.06)	0.017	4.83 ± 1.63	−0.57 (−0.83 to −0.31)	<0.001	0.914	0.126
Leg fat (kg)	10.10 ± 2.46	−0.93 (−1.1 to −0.1)	<0.001	10.68 ± 3.73	−0.18 (−1.66 to −0.71)	<0.001	0.148	0.515
Arm lean mass (kg)	4.60 ± 1.59	−0.06 (0.12 to 0.23)	0.480	5.28 ± 1.65	0,03 (0.09 to 0.15)	0.631	0.596	0.144
Leg lean mass (kg)	16.28 ± 3.12	−0.07 (−0.57 to 0.43)	0.770	15.48 ± 3.51	0.19 (−0.65 to 0.28)	0.406	0.181	0.601
Tot lean mass (kg)	52.15 ± 9.77	−0.49 (−1.33 to 0.35)	0.236	48.54 ± 9.53	−0.02 (−1.11 to 1.07)	0.972	0.545	0.611
Tot fat mass (kg)	36.11 ± 8.40	−3.81 (−4.80 to −2.82.8)	<0.001	37.05 ± 9.02	−3.72 (−5.23 to −2.20)	<0.001	0.770	0.820
Fat mass (%)	39.74 ± 7.50	−2.54 (−3.47 to −1.62)	<0.001	42.14 ± 7.98	−2.46 (−3.45 to −1.47)	<0.001	0.563	0.854
ALM (kg)	21.88 ± 4.64	0.01 (−0.59 to 0.56)	0.970	20.76 ± 5.10	0.16 (−0.68 to 0.36)	0.528	0.267	0.570
ALM/BMI	0.67 ± 0.15	0.14 (0.09 to 0.19)	<0.001	0.65 ± 0.17	0.03 (0.01 to 0.04)	0.008	0.531	0.382
Gait speed (s)	3.80 ± 1.13	−0.26 (−0.61 to 0.10)	0.141	3.60 ± 0.88	−0.12 (−0.56 to 0.31)	0.554	0.377	0.908
Chair stand (s)	12.50 ± 3.03	−3.24 (−4.56 to −1.93)	<0.001	10.32 ± 1.96	−1.46 (−2.10 to 0.82)	<0.001	0.173	0.270
SPPB (score)	10.94 ± 1.00	0.75 (0.29 to 1.21)	0.003	11.39 ± 0.78	0.39 (0.16 to 1.04)	0.030	0.321	0.528
Peak HGS (kg)	32.46 ± 8.20	2.67 (1.13 to 4.21)	0.002	33.66 ± 8.35	2.85 (0.16 to 5.54)	0.039	0.697	0.993
Peak HGS/BW (kg/kg)	0.35 ± 0.08	0.14 (0.04 to 0.24)	<0.001	0.38 ± 0.07	0.38 (0.04 to 0.24)	0.004	0.882	0.953

Abbreviations—BW: Body Weight; BMI: Body Mass Index; WC: Waist Circumference; FM: Fat Mass; LM: Lean Mass; GS: Gait Speed; SPPB: Short Physical Performance Battery; HGS: Handgrip Strength; RT: Resistance Training; EAAs: Essential Amino Acids.

**Table 3:** Maximal isometric torque at different knee angles after five months of intervention.

All Groups	RT (n = 16)		Within-Group PRE vs. POST	RT+EAAs (n = 18)		Within-Group PRE vs. POST	Between-Group PRE	Between-Group PRE vs. POST
Outcome	Baseline	Mean Change (95% CI)	*p* Value	Baseline	Mean Change (95% CI)	*p* Value	*p* Value	*p* Value
Optimal angle (°)	67.06 ± 7.13	−1.13	0.712	64.71 ± 8.59	1.62	0.435	0.472	0.741
		(−7.57 to 5.31)			(−2.66 to 5.91)			
Tmax (Nm)	178.14 ± 59.52	15.22	0.007	166.82 ± 44.00	10.41	0.171	0.530	0.532
		(4.87 to 25.57)			(−4.96 to 25.79)			
Tmax/kg (Nm/kg)	1.93 ± 0.54	0.28	<0.001	1.88 ± 0.43	0.20	0.027	0.752	0.420
		(0.16 to 0.40)			(0.03 to 0.38)			
30 MVC (Nm)	114.5 ± 34.72	11.06	0.173	108.78 ± 24.04	8.33	0.090	0.577	0.790
		(−5.42 to 27.54)			(−1.45 to 18.11)			
30 MVC/kg (Nm/kg)	1.24 ± 0.30	0.18	0.044	1.23 ± 0.26	0.15	0.017	0.922	0.713
		(0.01 to 0.36)			(0.03 to 0.26)			
60 MVC (Nm)	173.44 ± 56.73	14.13	0.065	161.94 ± 39.68	10.56	0.211	0.495	0.694
		(−0.96 to 29.21)			(−6.57 to 27.68)			
60 MVC/kg (Nm/kg)	1.88 ± 0.49	0.26	0.007	1.82 ± 0.38	0.20	0.046	0.713	0.581
		(0.08 to 0.43)			(0.00 to 0.40)			
75 MVC (Nm)	173.94 ± 61.70	8.88	0.123	162.83 ± 50.87	12,44	0.096	0.569	0.903
		(−2.70 to 20.45)			(−2.48 to 27.36)			
75 MVC/kg (Nm/kg)	1.89 ± 0.60	0.21	0.006	1.83 ± 0.52	0.23	0.011	0.770	0.988
		(0.07 to 0.35)			(0.06 to 0.40)			
90 MVC (Nm)	148.31 ± 48.25	5.56	0.185	134.44 ± 37.24	9.67	0.143	0.352	0.950
		(−2.96 to 14.09)			(−3.60 to 22.93)			
90 MVC/kg (Nm/kg)	1.61 ± 0.45	0.16	0.001	1.51 ± 0.37	0.18	0.014	0.503	0.957
		(0.98 to 0.25)			(0.04 to 0.32)			

Abbreviations—MVC: Maximal Voluntary Contraction; BM: Body Mass; Tmax: Maximal Torque at Knee Angle; RT: Resistance Training; EAAs: Essential Amino Acids.

## Data Availability

The datasets generated during and/or analyzed during the current study are not publicly available since this option was not included in the originally signed consent. Data are, however, available from authors upon reasonable request.

## Abbreviations

The following abbreviations are used in this manuscript:

RT	resistance training
EAA	essential amino acid
DO	dynapenic obesity
DXA	dual X-ray absorptiometry
1-RM	one-repetition maximum
SPPB	short physical performance battery
HGS	handgrip strength
ALM	appendicular lean mass
BMI	body mass index
T-max	maximal torque
ANCOVA	analysis of covariance
ANOVA	analysis of variance
